# Maya Healers' Conception of Cancer as Revealed by Comparison With Western Medicine

**DOI:** 10.1200/JGO.2015.001081

**Published:** 2016-01-27

**Authors:** Mónica Berger-González, Eduardo Gharzouzi, Christoph Renner

**Affiliations:** **MónicaBerger-González**, Swiss Federal Institute of Technology, ETH Zürich, Zürich; **ChristophRenner**, University Hospital Basel, Basel, Switzerland; and **EduardoGharzouzi**, Cancer Institute of Guatemala, Guatemala City, Guatemala

## Abstract

**Purpose:**

Cultural diversity in clinical encounters is common, yet mental constructions regarding cancer that influence expected treatment are poorly studied for indigenous people. We explored Maya healers' conceptions, diagnosis, and treatment of cancer to remedy this problem.

**Methods:**

In-depth structured interviews with 67 traditional Maya healers in Guatemala across Kaqchikel, Kiche', Mam, Mopan, and Q'eqchi' ethnolinguistic groups were conducted by using a transdisciplinary format. Analysis of qualitative data in categorized matrixes allowed for statistical examination of tendencies and the results were complemented by validation workshops with Maya representatives.

**Results:**

Maya classification of diseases has broad categories of malignant diseases including cancer. Specific Maya terms might equate to particular cancer types, which would open new avenues for research. Notions of malignancy and metastasis were expressed by healers as core characteristics of cancer, a disease believed to be both material and spiritual. Resolution of and/or treatment for cancer is based on restoring physical, mental, emotional, and spiritual equilibrium of the patient and extending that equilibrium to his larger social circle.

**Conclusion:**

Maya conceptions of cancer determine how traditional diagnostic tools are used and dictate treatment options that include the patient's social-spiritual support system. Official health care providers' understanding of these principles can improve implementation of culturally appropriate protocols that increase indigenous patients' compliance and reduce rates of treatment abandonment.

## INTRODUCTION

The ways in which social groups represent diseases differ from one society to another. Any understanding of an etiologic representation of a system of medical concepts needs to be grounded on the social-cultural conditions in which it is inscribed.^1^ Ethno-medicine refers to the study of medical systems or healing practices of cultural groups. It defines how well-being and suffering are experienced and interpreted bodily and socially within that structure.^2^ Biomedicine or Western medicine also needs to be understood as a cultural system^3,4^ to be compared with other systems without implicit value judgments.^5^ This is relevant in multicultural societies in which medical pluralism characterizes the health-seeking pathways of many patients.^6,7^ In such settings, Western physicians often face great challenges when implementing treatment for patients whose belief systems are rooted in worldviews different from their own (ie, as seen in a higher rate of treatment abandonment among indigenous patients^8^). This is at least partially related to language barriers and differing cultural representations of the etiology of disease and corresponding expectations for appropriate healing.^9,10^

In Guatemala, most of the Maya population (58%) live below the poverty line and have little access to official health care services.^11^ Many rely on traditional healers, which accounts for the coexistence of two medical systems that are characterized by inequity and are often in conflict.^12^

There are approximately 14,000 cancer cases per year in Guatemala,^13^ with an increase of 52% in incidence and 54% in mortality expected by 2025. Although there is interest in documenting cancer types that affect indigenous people worldwide,^14,15^ there are no data regarding cancer prevalence among the Maya of Guatemala. Instituto Nacional de Cancerología's (INCAN′s) Hospital Registry is Guatemala's only national reference, one not sensitive to ethnicity categories, and it is estimated to cover approximately 23% of the country's cancer cases.^16^

As the cancer burden increases, the national health care system will face an increase in referrals of indigenous patients. Understanding what cultural representations and expectations Maya medical practitioners have about cancer is key to determining culturally appropriate and medically effective disease management processes. Understanding cultural differences may reveal areas for cooperation between medical practitioners of both systems.

In Western medicine, classification of symptoms within known disease categories is achieved through a defined diagnostic process that relies on objective physical examinations and imaging technologies. The traditional healing process of the Maya, in existence for more than 2,000 years, is based on systematic observation and interpretation of symptoms, suffering, causes, effects, and responses.^17-21^ There are many models for Mesoamerican classification of diseases that provide a foundation for understanding Maya medicine,^22-31^ yet few contain cancer.^32^

This information gap was addressed in the Maya and Contemporary Conceptions of Cancer study. We focused on three basic questions concerning Maya medical epistemology: (1) What are the basic conceptions of Maya healers regarding cancer? (2) How do they diagnose cancer? and (3) How do they treat cancer? Here we report the understandings of tumors and cancer of 67 Maya traditional healers.

## METHODS

This study was conducted in Guatemala by using a transdisciplinary process^33,34^ between scientists from Europe (led by the Swiss Federal Institute of Technology, ETH Zürich), the United States, Guatemala (led by INCAN), and the Guatemala Maya Council of Elders. An extensive investigation was conducted from January 2011 to May 2013 to identify, select, and interview traditional Maya healers and validate collected data with five regional Councils of Elders (one per ethnolinguistic region: Kaqchikel, Kiche', Mam, Mopan, Q'eqchi'). The methodologic process is described in Berger et al^34a^ and is briefly explained here.

### Data Acquired by Interview

A sample of 67 Maya elders, 13 per ethnolinguistic group (Q'eqchi', 15), were interviewed in their traditional Maya language. The interview process followed a medical anthropology format enriched by members of the Western medical profession and later discussed and validated by the Guatemala Maya Council of Elders during two workshops. The interview was revised to make it culturally appropriate for obtaining in-depth medical knowledge from the Maya and was later translated into five Maya languages. The final interview guide had 127 questions organized into 11 sections (Data Supplement). The first questions were designed to discover intrinsic categories of Maya healers' classifications of diseases and then move progressively toward matching Western biomedical concepts of tumors and cancer. No mention of the term cancer was made until the interview's last section to avoid forced elicitation of biomedical terms that affect the Maya emic categories and explanatory models.^34b^ We use lowercase “cancer” throughout this article as the Maya term in which no biomedical evidence is available and “Cancer” with an initial capital letter C for the Western medical clinical definitions.

### Data Analysis and Statistical Evaluation

The interviews were conducted in 67 Maya towns and yielded more than 300 hours of recordings. Maya linguists transcribed and interpreted interviews into Spanish and created glossaries of Maya terms that could not be translated. Each regional council held validation workshops to revise results and produce a final synthesis that was later used to compare independent analyses of interviews by the Guatemalan academic team. This article presents healers' responses to sections 5, 6, 8, and 11 of the interview guide (Data Supplement). Data analysis was based on directed qualitative content analysis and data were later processed by using SPSS 19 (SPSS, Chicago, IL) software. M.B.-G. observed Maya treatments over 5 years prior to and during the study, which allowed deeper insight into Maya medical practice.

## RESULTS

### Maya Healers in the Sample

Of Maya healers in the study, 58.2% were male and 41.8% were female ranging in age from 33 to 83 years (mean, 57 years). More than 75% of the healers had practiced traditional medicine for more than 20 years; generations of healers have gained their knowledge from their elders almost exclusively by oral transmission. Ten medical specialties were recorded in the sample; 72% of healers identified themselves as Ajkum (herbalists), 57% as Ajq'ij (day-keeper, spiritual healers), and 12% as Iyom (female obstetricians). A single healer could have multiple specialties. Forty-six percent were illiterate, and less than 8% had more than a primary school education. Most (95.5%) lived in rural areas with varying degrees of remoteness. A more thorough description of the healers interviewed is presented elsewhere (Berger et al, manuscript submitted for publication).

### Maya Conception of Cancer and Tumors

Of the Maya healers interviewed, 51 (83.6% of those answering) had previously heard the term Cancer. More than half (51.7%) had treated patients coming from hospitals with a definitive Cancer diagnosis.

When asked to explain what they understood about the disease that Western physicians called Cancer, only 35.8% gave a definitive answer. A summary of the predominant characteristics expressed is that Cancer is a disease not easy to cure (79.2%), it usually starts within a person's body (58.3%), except when a small element comes from outside into the body such as through sexual intercourse or bad food (16.7%), and it spreads through the blood to many other parts of the body (54.2%). Cancer causes the flesh to rot (45.8%) or produces lumps of hardened flesh (16.7%). It advances with time, so that at first, there is no pain (37.5%), but when pain becomes almost unbearable (16.7%), it has worsened to an evil (malignant) state (25%). It is caused by many different things (100%). For emic perspectives, see the Data Supplement. In all, 71.9% of healers reported having seen patients with hard tissue lumps called tumors, whereas 69.6% reported diagnosing and treating this condition in different body areas (Data Supplement).

### Maya Classifications of Disease and Cancer

**Figure 1:**
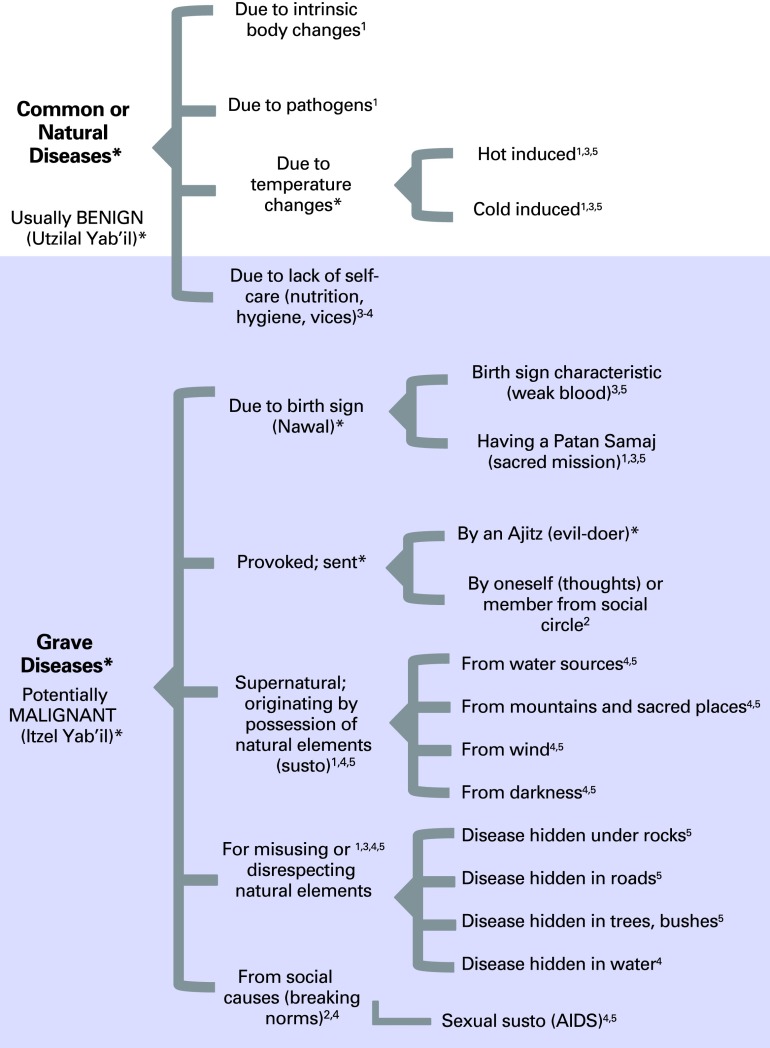
Classification of diseases according to the 67 Maya healers following a synthesis within each ethnolinguistic group. Classification subsystems are designated as follows: Kaqchikel (1), Kiche' (2), Mam (3), Mopan (4), and Q'eqchi' (5). Terms in bold and those followed by an asterisk refer to shared concepts across four groups (1,2,3,5). The gray area shows where cancer and malignant tumors can occur.

Maya healers provided complex taxonomies for classifying diseases, adhering to, and expanding prior descriptions.^29^ Figure 1 presents a graphic synthesis of the main disease classification system used by interviewed healers based on what the healers believed to be the origin of the disease. The underlying cosmologic assumptions of this system are presented elsewhere. The gray area emphasizes classifications in which cancer and tumors are more likely to occur, coinciding with the broader category of Itzel Yab'il (malignant disease), defined as disease leading to death if untreated. Maya emic views on such categories are presented in the Data Supplement.

Maya healers provided lists of diseases they treat along with their associated symptoms. Table 1 presents Maya terminology for inflammatory processes, tumors, and particular cancer types. The concept of hard tumors is easier to identify, yet the notion of a progressive disease that damages tissue and organs distinctly emerges.

**Table 1:**
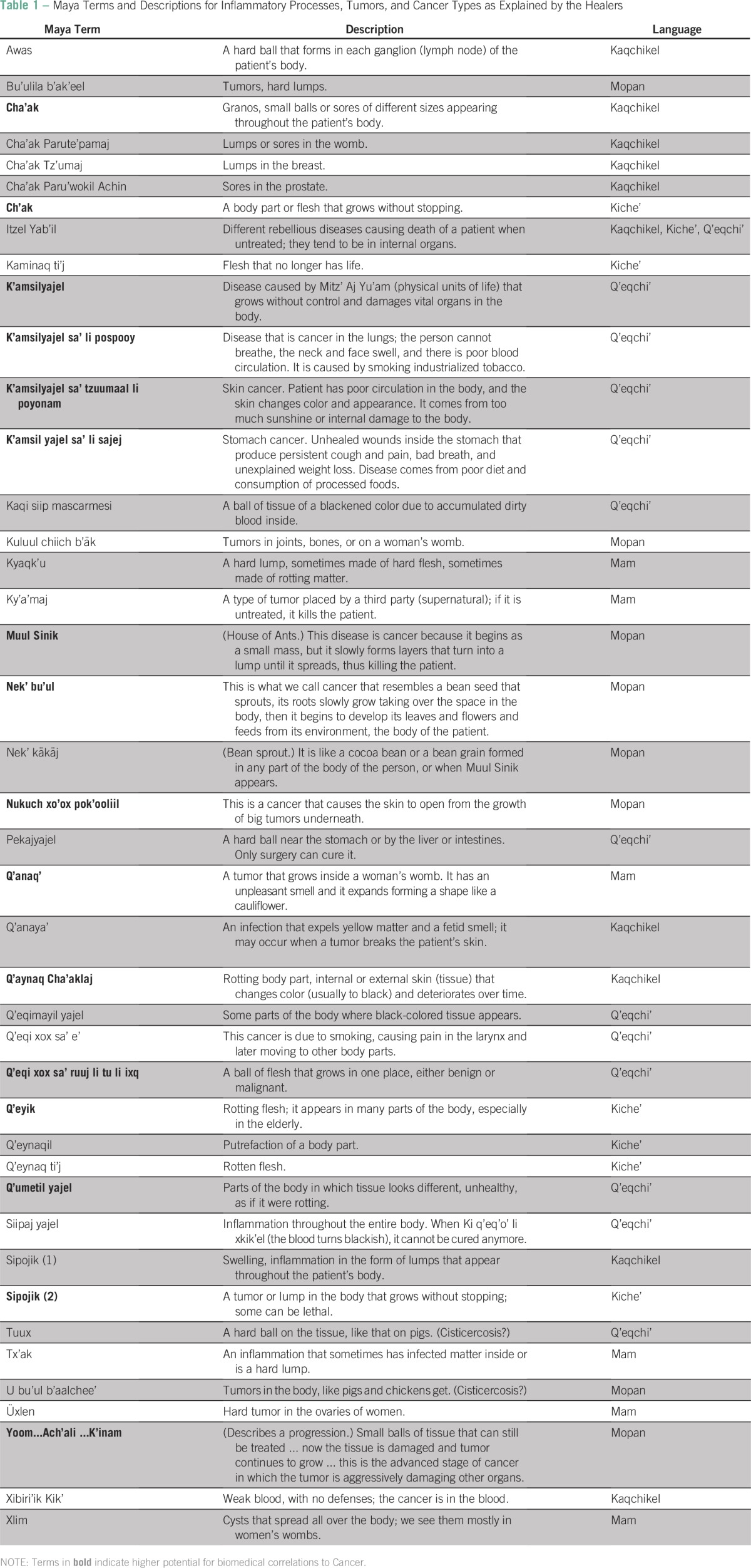
Maya Terms and Descriptions for Inflammatory Processes, Tumors, and Cancer Types as Explained by the Healers

When asked if the emic descriptions of tumors and malignancies (including cancer types) they provided were related to the Western scientific notion of Cancer, 52% of the healers answered positively. The concept of malignancy is mentioned as a core characteristic of cancer and bad tumors by 85% of healers and is described as a progression of the disease that causes death if untreated. Most diseases classified as Itzel Yab'il (Fig 1) have this characteristic.

Another essential characteristic given by 83.6% of healers for cancer and bad tumors is that cancer is mobile within the body. Only 21% of healers conveyed that cancer is contagious; most stated that it could not be transmitted to another person. Further exploration of the concept of cancer being contagious revealed that half of these healers related this to an understanding that cervical cancer can be passed to women via sexual intercourse, that cancer can be passed from mother to baby (ie, the mother passes the disease in the seed to her children [Qeq10]), or that cancer can be passed through contaminated food or water.

Maya healers' explanations of the causes of tumors and cancer are summarized in Table 2. Healers distinguished between material factors affecting the body and nonmaterial factors related to emotions, mental states, and spiritual belief.

**Table 2:**
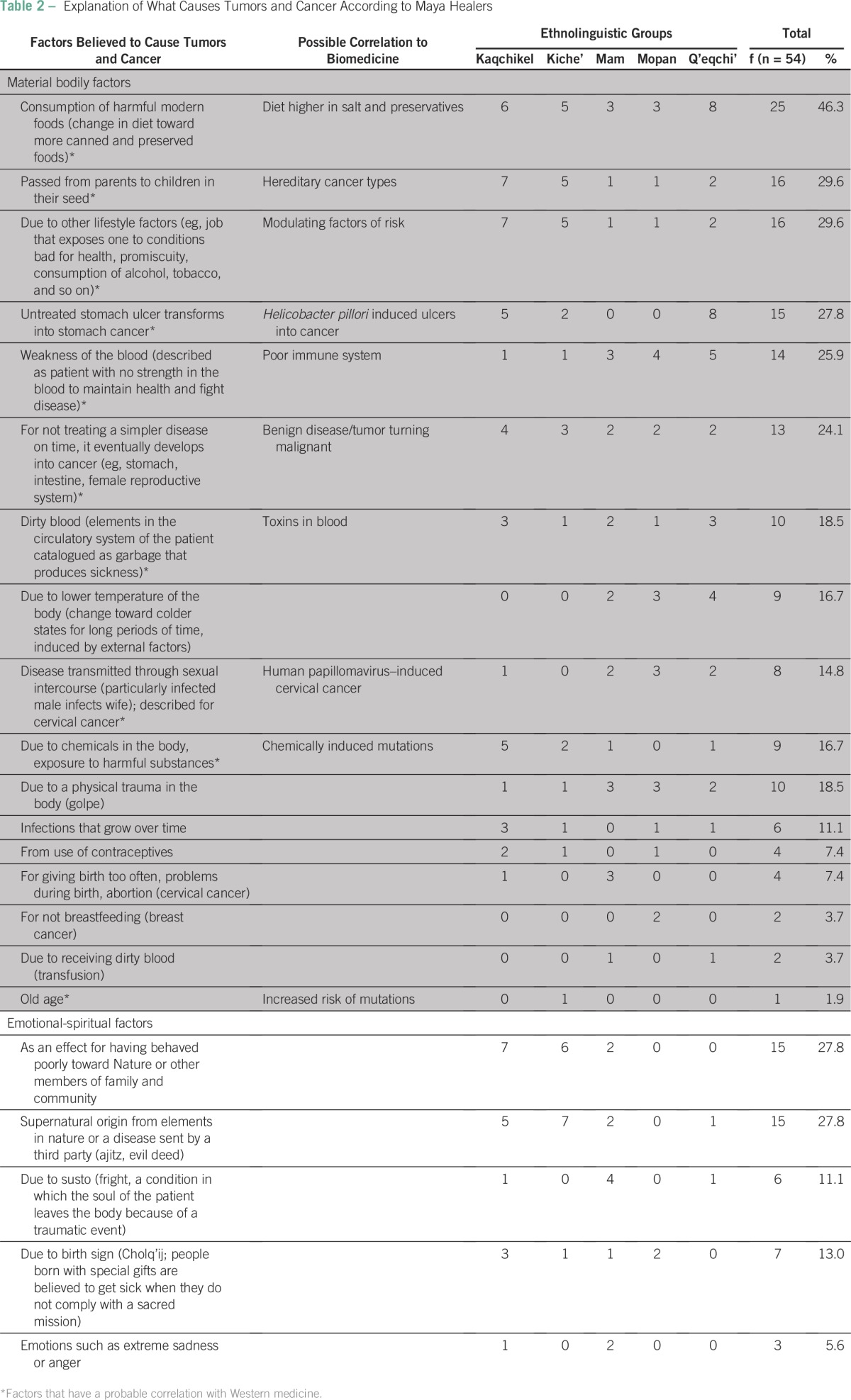
Explanation of What Causes Tumors and Cancer According to Maya Healers

The top five physical reasons given to explain the origin of cancer are consumption of modern foods that are considered harmful (46.3%), hereditary conditions (29.6%), lifestyle factors such as smoking or working with toxic substances (29.6%), untreated stomach ulcers (27.8%), and having weak blood (25.9%; understood as not being able to fight disease normally). The observation that 10 of 17 physical causes seem to correlate to Western knowledge about the etiology of cancer is remarkable (indicated with an asterisk in Table 2). The two most important nonmaterial causes of cancer and tumors are supernatural and take the form of either disease provoked by third parties or possession by elements in nature (27.8%) or behavioral conditions such as transgressions against the social or natural environment (27.8%). These seem to be more important to the Kaqchikel and Kiche' healers.

### Cancer Diagnosis in Maya Medicine

Diagnosis is preceded by prayer and by interviewing the patient to reconstruct the case. Methods used by Maya healers for diagnosing cancer are summarized in Table 3 divided into two primary groups: material and spiritual. Material methods relate to a patient's physical aspects, which are easily observed and measured. Spiritual methods cannot be verified by an independent observer.

**Table 3:**
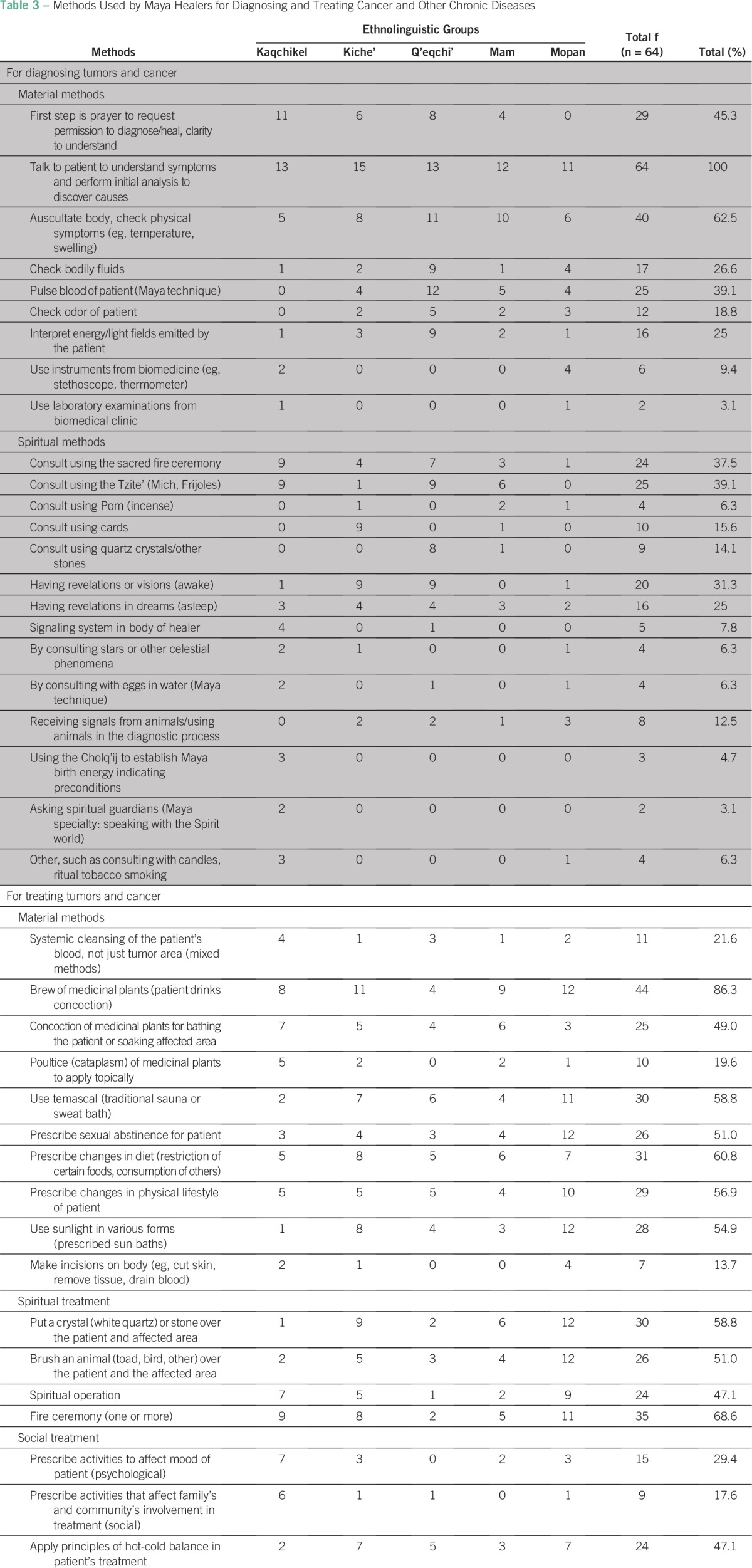
Methods Used by Maya Healers for Diagnosing and Treating Cancer and Other Chronic Diseases

The most commonly used diagnostic methods (62.5%) are physical auscultation and checking general bodily symptoms (fever, swelling, fluids), pulsing the blood of a patient (39.1%), consulting the spiritual dimension through the Tzite' (energetic divinatory reading) (39.1%), consulting the spiritual dimension through a sacred fire ceremony (37.5%), and experiencing revelations by one's spiritual guides or ancestors (31.3%). The last three are typical of Ajq'ij healers. Pulsing of blood is a well-described, complex Maya method^35^ requiring many years of training. When asked about the reliability of diagnosis, 34.9% of healers said it was possible to fail (reasons are provided in the Data Supplement), 27% said they had never been wrong, and 38.1% did not answer.

### Cancer Treatment in Maya Medicine

Fifty-one healers provided information on how they treat tumors and cancer (Table 3). Among material methods (mediated by an observable unit), medicinal plants ground up or prescribed as teas or tinctures were used by 86.5% of healers. Plants are also applied topically (19.6%) and used in sweat baths or hot baths (49%) when medicinal baths for skin cancer or detoxifying treatments are prescribed. A single healer's pharmacopeia of knowledge may include more than 400 plant species used to treat chronic disease. Ritual blessing of every step involved in healing, such as harvesting plants or preparing bandages, is a key characteristic of Maya medicine.

As part of treatment, 60.8% of healers prescribed changes in diet (emic accounts; Data Supplement); 56.9% prescribed changes in lifestyle, including regulating exercise, modifying hygiene habits, and banning smoking; and 51% prescribed sexual abstinence to “avoid dissipation of vital energy … needed to recuperate” (QEQ02). Sunlight is used by 54.9% of healers as sunbaths or with plant/clay ointments over affected body parts, showing possible equivalents to the principles of photodynamic therapy.^36^

Spiritual methods include the use of ritual sacred fire ceremonies (68.6%) and brushing animals (51%) or quartz/stones (58.8%) over the patient's body “to trap the disease, clean and harmonize the vital energy of the patient” (KAQ13). Spiritual surgeries, considered a divine gift, are used by 47.1% of healers. Social treatment includes counseling families and individuals to strengthen bonds between them and their community. Forty-five percent of healers indicated that they provided psychological-emotional support to patients and their larger social circle during treatment.

On average, each healer used seven treatment methods (Table 3) per patient, with 98% always combining material and social-spiritual practices. Almost half the healers apply principles of hot-cold balance^37,38^ to create internal-external equilibrium (ie, selecting cold plants for treating a hot disease such as stomach cancer).

## DISCUSSION

Maya healers' experience with patients who have Cancer is unequivocal and provides ample opportunity to observe, study, diagnose, and treat the disease. Perhaps the healers' proximity to patients with Cancer who have abandoned treatment in the public health system has presented an opportunity for cross-cultural definitions to emerge.

The Maya concept of Cancer as revealed by the Maya and Contemporary Conceptions of Cancer study shows that there are aspects that correspond to modern oncology, as seen in equivalencies in notions of malignancy and metastasis and more than half the explanations about its causes (Table 2). Likewise, Maya descriptions of contagion relate to Western conceptions of hereditary and virus-induced Cancer types (such as human papillomavirus for cervical cancer). It also shows that Cancer is perceived as both a material and an emotional/spiritual disease, as observed in healers' belief in spiritual and social causes for its appearance in patients (Table 3). Maya healers state that patients must assume great responsibility for having contributed to the emergence of Cancer. This is in contrast to most modern oncologists who treat the disease mostly as a matter of chance with risk modulators.^39^ Maya healers assign co-responsibility to their patients from the onset of the diagnosis and extend it to treatment and healing. Patients are not passive bystanders but must be active participants in overcoming the disease. The notion that Cancer stems from the internal loss of equilibrium within the patient's body, mind (ideas/thoughts), and heart (feelings), as well as from the external loss of equilibrium with the larger socioecological environment and spiritual realm is a core characteristic of Maya healers' epistemic system. This conceptual model determines specific methods used to diagnose and treat chronic disease.

Although there is no 1:1 correlation between any specific Maya terms for their diagnosed diseases and Cancer, our data provide evidence for local taxonomies of disease that may correspond to certain Cancer types. The broad categories used by most Mayas, such as that of itzel yab'il, portray a notion of malignancy in which Cancer and other chronic pervasive diseases are included. An equivalent to the medical Western definition of tumors is more clearly expressed by Maya healers who separate those designated as benign (utzilal) from those designated malignant (itzelal).

Most Maya healers rely on a combination of material and spiritual methods during the cancer diagnostic process deemed necessary to understand all aspects of the disease. More than 17 different spiritual diagnostic methods were reported versus eight material ones, showing another important characteristic of Maya medicine: it relies predominantly on its belief system, which ascribes great importance to the spiritual world of ancestors and supernatural energies explained elsewhere.^31,35,40-42^ Little evidence has been reported for using biomedical concepts and tools. Ancient physical diagnostic methods such as blood pulsing or interpretation of emissions of light-energy from the body are still used. An ongoing study between Maya healers and INCAN oncologists may shed light on the accuracy of traditional Maya diagnostic methods.

While treating cancer, Maya healers use a variety of physical, spiritual, and social methods aimed at restoring balance to a patient's body, thoughts, feelings, and behavior and in his immediate social network. Treatment is therefore holistic and is oriented toward treating the causes of the disease and restoring well-being in the patient and does not focus on treating symptoms or specific body parts.^43,44^ Although grouped into 17 categories for clarity, treatment methods are complex as seen in more than 40 specific tools and processes applied by healers according to their specialty and healing tradition. Phytotherapy is the treatment method most relied on by healers, followed by sacred fire ceremonies, which again reveals the Maya complementarity between material and nonmaterial (spiritual) healing processes.

Healing treatments observed by M.B.-G. show some disparity between the emics of mental life and the etics of behavioral stream,^45^ because healers who were interviewed simplified ideas and descriptions of healing procedures when talking in abstract terms that did not match the complexity observed in real-life situations.

Opportunities for comparing Maya and Western medicine may increase because complementary medicine (including plant medicine) is increasingly applied in the biomedical setting, which creates new opportunities for common research.

In conclusion, Maya conceptions of cancer and associated methods for its diagnosis and treatment present an interesting case of the interface between biology and culture. Although the physical experience of cancer may be the same for patients regardless of their ethnolinguistic origin, the sociocultural experience of this disease is shaped by conventions of what is normal and what is pathologic and by the aspects deemed important or valid in treatment in each culture. Maya medicine adherents are influenced by the explanatory models of Maya healers, which creates expectations of what ideally should be done in a healing setting. These expectations are often ignored and consequently disregarded by Western doctors in hospitals and clinics, which often has a negative effect on these patients. In INCAN, 30% of patients never start treatment after diagnosis, and 30% of those who start treatment do not finish,^16^ a trend at least partially related to the patients' cultural disagreements with procedures used by the hospital's staff. In countries with multicultural traditions such as Guatemala, Western medical service providers would benefit from knowing more about their patients' mental medical models and from understanding what adaptations are necessary to support a more efficient and successful treatment process (eg, spiritual blessing of chemotherapy). A key implication gleaned from this study is that the Hippocratic oath committing physicians to serve patients to the best of their ability may require a major effort on the part of those physicians to transport themselves out of their own comfort zone of medical paradigms and attempt to learn about other perspectives that shape the beliefs and expectations of indigenous patients.
